# Patient selection for accelerated partial breast irradiation (APBI) after breast-conserving surgery: Updated evidence-based recommendations of the Groupe Européen de Curiethérapie-European Society for Therapeutic Radiology and Oncology (GEC-ESTRO) Breast Cancer Working Group

**DOI:** 10.1016/j.ctro.2026.101170

**Published:** 2026-04-17

**Authors:** Csaba Polgár, Cristina Gutierrez-Miguelez, Olivera Ivanov, Viktor Smanykó, Magdalena Stankiewicz, Irene Martínez, Kristina Lössl, Jose-Luis Guinot, Tibor Major, Jean-Michel Hannoun-Levi, Vratislav Strnad, on behalf of the GEC-ESTRO Breast Cancer Working Group

**Affiliations:** a National Institute of Oncology, Center of Radiotherapy, Budapest, Hungary; b Semmelweis University, Department of Radiotherapy, Budapest, Hungary; c Hospital del Mar, Department of Radiation Oncology, Barcelona, Spain; d Institut Catalá d’ Oncologia, Department of Radiation Oncology, Barcelona, Spain; e Oncology Institute Vojvodina, Department of Radiation Oncology, Sr Kamenica, Serbia; f Faculty of Medicine, University of Novi Sad, Novi Sad, Serbia; g Maria Sklodowska-Curie National Research Institute of Oncology, Brachytherapy Department, Gliwice, Poland; h Hospital Universitario de Navarra, Department of Radiation Oncology, Navarra, Spain; i Inselspital, Bern University Hospital, University of Bern, Department of Radiation Oncology, Switzerland; j Fundacion Instituto Valenciano de Oncologia, Department of Radiation Oncology, Valencia Spain; k Centre Antoine-Lacassagne, Department of Radiation Oncology, University Côte d’Azur, Nice, France; l University Hospital Erlangen, Department of Radiation Oncology, Erlangen, Germany

## Abstract

**Purpose:**

To update recommendations on patient selection criteria for accelerated partial breast irradiation (APBI) based on available clinical evidence supplemented by expert opinions.

**Methods and materials:**

Between 2010 and 2024, a systematic search of the PubMed, Medline, Scopus and Cochrane database identified 618 articles using the keywords “accelerated partial breast irradiation” and “APBI”. This search was complemented by reviewing the reference lists of articles and manual reviewing of relevant conference abstracts and book chapters. Of these, ten prospective randomized clinical trials and seven retrospective comparative studies with a minimum median follow-up time of five years were identified. The authors reviewed the clinical evidence published on APBI, supplemented it with relevant clinical and pathological studies on breast-conserving therapy, and then formulated the recommendations presented in this manuscript.

**Results:**

Based on published new clinical evidence, the GEC-ESTRO Breast Cancer Working Group recommends two categories as guidelines for selecting patients eligible for APBI: (1) low-risk group representing good candidates for APBI including patients ageing > 40 years with unifocal or multifocal within 2 cm, pTis,T1-2 (≤30 mm) pN0 or pN1mi, all histology types of breast cancer without the presence of an extensive intraductal component (EIC), without extensive lympho-vascular invasion (LVI) and with negative surgical margins for invasive tumors (≥2 mm for DCIS), (2) high-risk group, for whom APBI is considered contraindicated including patients with BRCA 1–2 mutations or ageing < 40 years; having positive margins for invasive tumor (<2 mm for DCIS), and/or multicentric or large (>30 mm), and/or triple negative tumours, and/or EIC positive, and/or extensive lympho-vascular invasion (LVI) or macrometastatic positive lymph nodes (≥pN1a) or unknown axillary status (pNx).

**Conclusions:**

Based on emerging clinical evidence, the 2010 GEC-ESTRO APBI patient selection criteria can be significantly expanded, meaning that in the future, more patients may receive APBI as a part of routine clinical practice.

## Highlights

• The GEC-ESTRO Breast Cancer Working Group (BCWG) published recommendations on APBI patient selection in 2010.
• Based on the emerging evidence the GEC-ESTRO BCWG represents their updated recommendations on APBI patient selection.
• The original 2010 GEC-ESTRO APBI patient selection criteria could be significantly widened, allowing more patients to be treated with APBI as a part of daily clinical practice in the future.

## Introduction

Since the 1980-s, breast-conserving surgery (BCS) followed by whole breast irradiation (WBI) consisting of five weeks of daily external beam radiotherapy (RT) with or without additional irradiation to the tumour bed has become the standard of care for the treatment of early-stage breast carcinoma [1], [2], [3]. Later, moderately and ultra-hypofractionated WBI over one to three weeks proved to be non-inferior to conventionally fractionated (2 Gy/fr.) WBI [4], [5]. At the same time, in the 1990s, it was questioned whether WBI was necessary for all patients after BCS, and several centers investigated the feasibility and efficacy of accelerated partial breast irradiation (APBI) in prospective phase 2 and 3 clinical trials [6], [7], [8], [9], [10]. The results of these clinical trials showed that APBI using with conservative patient selection, appropriate target volume definition, and irradiation technique yields similar results to those achieved with standard WBI. Based on these evolving clinical evidence, international scientific societies and groups have gradually published guidelines for APBI patient selection [11], [12], [13], [14], [15]. Among these, the Breast Cancer Working Group (BCWG) of the Groupe Européen de Curiethérapie-European Society for Therapeutic Radiology and Oncology (GEC-ESTRO) in 2009, also formulated recommendations on patient selection criteria for the use of APBI outside of prospective clinical trials [12]. The original GEC-ESTRO recommendations identified three categories to serve as guidelines for selecting patients suitable for APBI: (1) a low-risk group for whom APBI outside of a clinical trial was an acceptable treatment option (so-called green-zone for APBI); (2) a high-risk group for whom APBI has been considered contraindicated (so-called red-zone for APBI); and (3) an intermediate-risk group for whom APBI has been considered acceptable only in the context of prospective clinical trials (so-called grey-zone for APBI). For the latter group of patients, those in the so-called grey-zone (e.g. patients with >40 to 50 years of age, or invasive lobular carcinoma, or close (but clear) surgical margins etc.) there was still small amount of clinical evidence available at that time to support the use of APBI in routine clinical practice. Other previously published guidelines on APBI have also faced uncertainties regarding how to identify “good candidates” for APBI, as there was insufficient evidence regarding the inclusion or exclusion of specific subgroups of breast cancer patients, and the question remained open: whether APBI can be applied to a broader patient population?

However, over the past 15 years, clinical evidence has emerged and matured significantly. Therefore, the GEC-ESTRO BCWG aims to update recommendations on patient selection criteria for the use of APBI.

## Material and methods

**Fig. 1:**
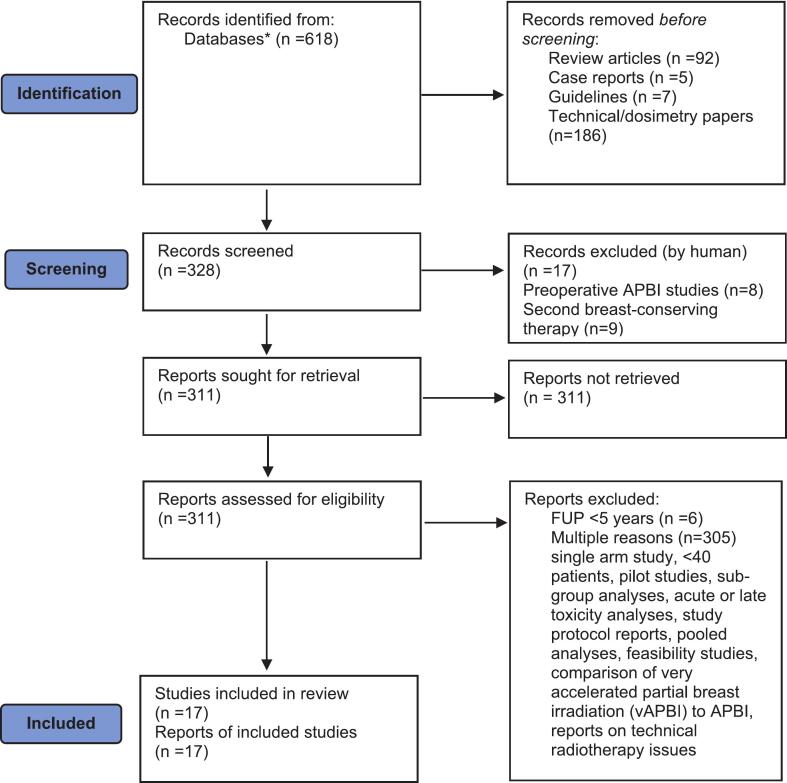
PRISMA flowchart diagram for systematic review. *Abbreviations:* APBI  = accelerated partial breast irradiation; FUP  = follow-up period.

A systematic search of the PubMed, Medline, Scopus and Cochrane database using the keywords “accelerated partial breast irradiation” and “APBI” was performed. The analyzed literature was dated from 2010 onwards, including publications with clinical evidence obtained from prospective randomized trials, prospective and retrospective comparative observational studies with a minimum median follow up of five years, with at least 40 patients enrolled in the study, and published in English language. Three investigators (OI, IM, and VS) independently conducted a systematic search on the topic of APBI. This search was complemented by reviweing the reference lists of articles and manual reviewing relevant conference abstracts and book chapters. Google scholar and manual searches were used to analyze articles for grey zone. The date of last query was 28th of December, 2024. Between 2010 and 2024, a total of 618 articles were identified, and after excluding review articles (n = 92), case reports (n = 5), guidelines (n = 7), and technical/dosimetric papers (n = 186), 328 original publications remained. Articles dealing with preoperative APBI (n = 8) and second breast-conserving therapy (n = 9) were also excluded. Among the remaining 311 original articles, ten prospective randomized clinical trials and seven non-randomized comparative studies with more than 40 patients, and a minimum median follow-up time of five years comparing the efficacy of APBI and WBI were identified. Most of the 311 trials were excluded from the final analysis for several reasons, including single-arm or pilot trials, less than 40 patients included, sub-group analyses, acute or late toxicity analyses, study protocol reports, pooled analyses, feasibility studies, comparison of very accelerated partial breast irradiation (vAPBI) and APBI, or reports on technical radiotherapy issues. Only 4 studies were excluded because their follow-up period was less than 5 years, but they met all other criteria. Six reviewers screened all records and reports, and worked independently of each other. Each reviewer collected data from reports on selected risk factors, and finally another reviewer analysed all of the collected data. Fig. 1 shows the PRISMA flowchart, which illustrates the entire process of study selection, including the exclusion criteria applied at each stage. To avoid any potential heterogeneity in the study results, we excluded the use of subgroup analyses and metaregression analyses. The authors reviewed the published clinical evidence on APBI, analyzed the inclusion criteria and other relevant clinical and pathological studies of breast-conserving therapy (BCT). Quality assessment of the included studies was performed using Cochrane Risk of Bias 2.0 for randomized trials [16]. For each randomized trial, the risk of bias was assessed with regard tothe randomization process, deviations from the intended interventions, missing outcome data and the measurement of the outcome, as well as the selection of the reported results. For all ten randomized trials, low risk of bias for all domains was estimated by two independent reviewers. For non-randomized studies included in the final analysis, ROBINS I risk of bias was assessed by two independent reviewers [17]. Low risk of bias was estimated for all seven studies regarding bias due to confounding, selection of the participants, classification of interventions (with deviations) due to the missing data and outcome measures, and bias in selection of the reported results. The recommendations presented in this manuscript were developed during a series of consensus conferences based on these reviews and analyses.

### Updated clinical results of prospective randomized APBI clinical trials

The original 2009 GEC-ESTRO BCWG recommendations on APBI patient selection were based on the results of multiple prospective non-randomized phase 2 clinical trials, and the 5-year results of the Budapest Phase 3 trial [12]. During the past 15 years, the 20-year update of the Budapest trial and the results of further nine randomized APBI clinical trials with follow-up periods of five to ten years have been published, confirming that APBI delivered with brachytherapy or external beam radiotherapy was non-inferior to standard WBI [18], [19], [20], [21], [22], [23], [24], [25], [26], [27], [28], [29], [30] (Table 1.). However, the situation is different in the case of intraoperative radiotherapy (IORT) used for APBI [28], [29], [30]. The ELIOT (electron intraoperative radiotherapy) trial using a single-shot electron intraoperative radiotherapy confirmed a higher rate of ipsilateral breast tumour recurrence (IBTR) in the ELIOT group than in the WBI group with a hazard ratio of 4.62 [28]. With regard to the TARGIT-A (targeted intraoperative therapy) trial, which used a 50 kV X-ray device for APBI, no reliable conclusions can be drawn due to significant shortcomings in the study design and controversial publications on the subject [29], [30]. Unfortunately, no statistical analysis was ever conducted following a follow-up period of sufficient duration covering the entire patient population, and the statistical methods used have been seriously criticized by experts in breast cancer radiotherapy and statistics [[31], [32], [33]]. Therefore, based on the available clinical evidence obtained from prospective randomized APBI clinical trials for selected early-stage patients treated with BCS, postoperative partial breast irradiation delivered with interstitial or intracavitary brachytherapy (BT), three-dimensional conformal radiotherapy (3D-CRT) or intensity modulated radiotherapy (IMRT) are the only valid treatment options [18], [19], [20], [21], [22], [23], [24], [25], [26], [27].

**Table 1:** Results of prospective phase III PBI trials published between 2010 and 2024.

Trial	Study period	PBI technique	Patient N^o^	Median FUP (y)	LR PBI (%)	LR WBI (%)	p-value
Budapest [18]	1998–2004	HDR BT/ELE	258	18.2	9.6*	7.9*	0.59
GEC-ESTRO [25]	2004–2009	HDR/PDR BT	1.184	10.4	3.51†	1.58†	0.074
NSABP B-39 [22]	2005–2013	3D-CRT/HDR BT	4.125	10.2	4.6†	3.9†	NS
Univ. Florence [20], [21]	2005–2013	IMRT	520	10.7	3.7†	2.5†	0.40
ELIOT [28]	2000–2007	IORT	1306	12.4	8.1†	1.1†	<0.0001
RAPID [23]	2006–2011	3D-CRT	2.135	8.6	3.0‡	2.8‡	NS
Barcelona [26]	NR	3D-CRT	102	10.3	2.0	2.0	NS
IMPORT-LOW [19]	2006–2011	IMRT	2.016	10.0	3.0	2.8	NS
TARGIT-A [29]	2000–2012	IORT	2.298	8.6	2.36	0.99	0.28
DBCG [27]	2009–2016	3D-CRT	865	7.6	3.1	1.7	0.30
**All studies**	**1998–2016**	**–**	**14.809**	**5–18.2**	**0–9.6**	**0–7.9**	**–**

*Abbreviations*

FUP  = follow-up period; LR  = local recurrence; PBI  = partial breast irradiation; WBI  = whole breast irradiation; HDR BT  = high-dose-rate brachytherapy; ELE  = electron; PDR  = pulsed dose rate; 3D-CRT  = three-dimensional conformal radiotherapy; IMRT  = intensity modulated radiotherapy; IORT  = intraoperative radiotherapy; NR  = not reported; NS  = not significant. *20-year actuarial rate; †10-year actuarial rate; ‡8-year actuarial rate; (All other data represent 5-year actuarial rates).

### Updated clinical results of non-randomized comparative APBI studies

Over the past 15 years, the results of seven non-randomized studies comparing the efficacy of APBI to WBI have been published [34], [35], [36], [37], [38], [39], [40] (Table 2). In line with phase 3 APBI trials, in five out of seven studies, the IBTR rates after APBI were similar to those achieved with WBI [34], [35], [36], [39], [40]. In one of the two negative retrospective comparative studies, intraoperative electron therapy was used for APBI, and the results were similar to the findings of the ELIOT phase 3 trial, which favoured WBI over APBI delivered with a single intraoperative shot of electrons [37]. In another comparative study conducted at a single institution, investigators from Evanston reported significantly higher local recurrence (LR) rate after APBI delivered with external beam irradiation (3D-CRT or IMRT) [38].

**Table 2:** Results of retrospective comparative PBI trials published between 2010 and 2024.

Trial	Study period	PBI technique	Patient N^o^	Median FUP (y)	LR PBI (%)	LR WBI (%)	p-value
St. Chiara H., Trento [37]	2000–2010	IORT ELE	516	13	12.7* 7.9^†^	5.0*4.1^†^	0.02
W. Beaumont H. [34]	1992–2001	HDR BT	398	12	3.8^‡^	5.0^‡^	0.40
W. Beaumont H. [39]	1983–2011	LDR/HDR BT	548	7.8	4.2^†^	3.7^†^	0.11
Evanston [38]	2002–2012	3D-CRT/IMRT	580	8	6.2^§^	1.0^§^	0.030
Saint Louis [36]	2002–2007	HDR BT	296	5	3.0	3.8	0.902
Cadiz [35]	2008–2019	HDR BT	76	6	0	0	NS
Taichung [40]	2014–2019	IOEB	732	5.1	2.7	2.2	0.72
**All studies**	**1992–2019**	**–**	**3.146**	**5–13**	**0–12.7**	**0–5.0**	**–**

*Abbreviations*

FUP  = follow-up period; LR  = local recurrence; PBI  = partial breast irradiation; WBI  = whole breast irradiation; IORT  = intraoperative radiotherapy; ELE  = electron; HDR  = high-dose-rate; LDR  = low-dose-rate; BT  = brachytherapy; 3D-CRT  = three-dimensional conformal radiotherapy; IMRT  = intensity modulated radiotherapy; IOEB  = intraoperative electronic brachytherapy; NS  = not significant. *15-year actuarial rate, ^†^10-year actuarial rate; ^‡^12-year actuarial rate; ^§^crude rate; (All other data represent 5-year actuarial rates).

Jacobs et al. [41] recently reported the 5-year outcome of a two-armed prospective multicenter cohort study conducted at three Dutch medical centers, in which APBI performed with intraoperative electron therapy (n = 268) and external beam therapy (3D-CRT or IMRT; n = 207) was compared. In the per-protocol population, the cumulative incidence of IBTR at five years was 11.9 after IORT and 4.3% (p = 0.005) after external beam APBI.

### Update on patient-, tumour- and treatment-related factors affecting decision-making in patient selection for APBI

#### Patient age

Young age has been shown to be a significant negative prognostic factor for IBTR [3]. Most series reported an increased breast failure rate when different age cut-offs were applied. In the previous GEC-ESTRO recommendations, patients >50 years were selected as good candidates for APBI, while patients between ages of 40 and 50 were classified as being in the intermediate-risk group, with a conclusion that further prospective studies are needed to justify the use of APBI in this age group [12].

Recent results from phase 3 randomized APBI clinical trials conducted over the past 15 years demonstrate that in patients aged 40–50 partial breast irradiation is not associated with a significantly higher risk of IBTR (Table 3) [18], [22], [24]. Vicini et al. [22] analyzed subgroups defined based on risk factors in the NSAPB B-39/RTOG0413 trial, which yielded results for the the largest cohort of patients <50 years (n = 1.622 pts.). They reported that there was no difference in treatment efficacy among the subgroups, including premenopausal patients (n = 1.588). The 10-year cumulative incidence of IBTR for premenopausal patients was 6.4% vs. 4.8% in the APBI and WBI groups, respectively, with a hazard ratio of 1.47 (95% CI: 0.93–2.34) and with a p-value of 0.28 for interaction. Polgar et al. [18] reported the 20-year results of the Budapest trial, in which they compared APBI with WBI. Age ≤ 40 years was a significant predictor for LR. However, among patients aged 41–50, there was no significant difference in LR between the two treatment modalities (see Table 3.). Investigators of the GEC-ESTRO trial reported that among women under 50 years of age, the 5-year cumulative incidence of IBTR following WBI was 2.3%, whereas following APBI it was only 1.1%, and the absolute risk of LR was not associated with either age or treatment arm [24]. Similar results were confirmed at ten-year follow up [25]. In the RAPID trial at random assignment, patients were stratified for age <50 vs. ≥50 years [23]. After median follow-up of 8.6 years, the authors did not report any age-related differences in the results. The TARGIT-A trial included 216 (9.4%) patients under the age of 50, and according to the results, the long term efficacy was similar, and there were no significant differences based on patient age in terms of LR, mastectomy free survival, distant disease free survival, overall survival, and breast cancer mortality that depends on patients’ age [30]. On the other hand, in an analysis of phase 2 prospective studies comparing APBI and WBI, three studies reported that being under 50 years of age was associated with a significantly higher risk of IBTR [42], [43], [44]. In the German-Austrian trial the conclusion was that patients <50 years of age should be excluded from APBI protocols [42]. When comparing patients with an age <50 years (49/274) vs. ≥50 years (225/274), the 5-year LR rate was 7.5% and 1.1%, respectively (p = 0.030). Vicini et al. [43] reported that a young age <50 years and positive margins were significantly associated with IBTR in cases of ductal carcinoma in situ (DCIS) treated with MammoSite balloon BT, but age did not have a significant effect on LR in patients with invasive breast cancer. In the PROMIS registry trial evaluating patients treated with multicatheter high-dose-rate (HDR) BT, multivariate analysis showed that being under 50 years of age was associated with a higher risk of LR, however, this subgroup analysis did not distinguish between very young (≤40 years) and young (>40 to 50 years) patients [44]. Other small-scale prospective, non-randomized series were unable to draw clear conclusions regarding the effect of age on treatment outcomes, as the sample sizes of young women were small. We believe, that the weight of evidence from large-scale prospective phase 3 studies far outweighs the controversial observations from small-scale phase 2 trials. Therefore, based on the latest results from randomized, controlled APBI trials, patients over the age of 40 can be considered suitable candidates for APBI.

**Table 3:** Local recurrence after APBI versus WBI for patients aged <50 years.

Trial	Type of study	Median FUP (months)	PBI technique	Patient N^o^	LR % (n)		p-value
					PBI	WBI	
Budapest [18]	Prospective phase 3	18.2	HDR BT/ ELE	51	12.9%* (3/26)	8.3%* (1/25)	NS
NSABP B-39 [22]	Prospective phase 3	10.2	HDR BT/3D-CRT	1.588^α^	6.4%^†^ (30/780)	4.8%^†^ (47/808)	0.28
GEC-ESTRO [24]	Prospective phase 3	6.6	HDR/PDR BT	182	1.1%^‡^ (1/91)	2.3%^‡^ (2/91)	0.55
German-Austrian [42]	Prospective phase 2	5.3	HDR/PDR BT	49	7.5%^‡^ (4/49)	-	0.03^§^
All studies				1.870	4.0% (38/946)	5.4% (50/924)	

*Abbreviations*

FUP  = follow-up period; LR  = local recurrence; PBI  = partial breast irradiation; WBI  = whole breast irradiation; HDR BT  = high-dose-rate brachytherapy; ELE  = electron; PDR  = pulsed dose rate; 3D-CRT  = three-dimensional conformal radiotherapy; *20-year actuarial rate; ^†^10-year actuarial rate; ^‡^5-year actuarial rate; ^§^p-value compared to older (≥50 years) patients; ^α^No. of premenopausal patients (all patients aged >18 years were eligible).

#### Invasive lobular carcinoma (ILC) and lobular carcinoma in situ (LCIS)

For decades, ILC was considered a relative contraindication for breast conservation, due to its multifocality and diffuse pattern of spreading [45]. However, there was no significant difference between lobular and non-lobular carcinomas in the incidence and site of in-breast failure relative to the location of the primary tumour [46], [47], [48], [49], [50], [51], [52]. However, in 2009 there was insufficient clinical evidence to support the use of APBI for the treatment of breast cancer outside the context of prospective clinical trials [12].

The new clinical evidence published over the past 15 years are summarized in Table 4. Among the 274 patients enrolled into the German-Austrian phase 2 APBI study, 45 patients (16%) had ILC, and he 5-year LR rate in patients with ILC did not differ significantly from that in patients with other histological types [42]. In the APBI arm of the GEC-ESTRO phase 3 trial, 85 out of 633 patients (13%) had ILC, and there was no significant difference in LR rate compared to all other patients having non-ILC [24]. In other prospective APBI trials, only a small number of patients with ILC was treated [20], [53], [54], [55], [56]. Only the retrospective comparative study of Mills et al [56] reported a significantly higher rate of LR in patients with ILC treated with APBI. Recently, Braunstein et al [57] published the largest series of APBI patients with ILC: three out 132 patients (2.3%) treated with external beam APBI developed LR yielding a 5-year cumulative LR rate of 3%.

**Table 4:** Local recurrence after APBI in patients with invasive lobular carcinoma versus other histologies.

Trial/Author	Type of study	Median FUP (mos)	Technique	N^o^ of patients	Histology		N^o^ of LR according to histology		Higher RR for ILC?
					Other ca.	ILC	Other ca.	ILC	
GEC-ESTRO [24]	Prospective phase 3	79	PDR/HDR BT	633	548 (87%)	85 (13%)	6 (1.1%)	3 (3.5%)	No (p = 0.19)
German-Austrian [42]	Prospective phase 2	64	PDR/HDR BT	274	229 (83.6%)	45 (16.4%)	6 (2.6%)	1 (2.4%)	No (p = NS)
Florence Univ. [20]	Prospective phase 3	60	IMRT	260	239 (91.9%)	21 (8.1%)	NR	NR	No (p = NR)
Braunstein LZ [57]	Prospective database, retrospective analysis	39	3D-CRT/ IMRT	1.248	1.116 (89%)	132 (11%)	NR	2 (3%)	No (p = NR)
Sumodhee S [53]	Prospective database, retrospective analysis	97	HDR BT	109	103 (94.5%)	6 (5.5%)	2 (1.9%)	0 (0%)	No (p = NR)
Geary RL [54]	Prospective	120	HDR BT	62	59 (95.2%)	3 (4.8%)	4 (6.7%)	0 (0%)	No (p = NR)
Genebes C [55]	Prospective	61	HDR BT	70	67 (95.7%)	3 (4.3%)	1 (1.4%)	0 (0%)	No (p = NR)
Mills MN [56]	Retrospective	60	HDR BT	946	878 (92.8%)	68 (7.2%)	14 (1.6%)	4 (5.9%)	Yes (p = 0.008)
All studies					3.239 (89.9%)	363 (10.1%)	33 (1.0%)	10 (2.8%)	

*Abbreviations*

APBI  = accelerated partial breast irradiation; WBI  = whole breast irradiation; FUP  = follow-up period; ILC  = invasive lobular carcinoma; LR  = local recurrence; RR  = relative risk; HDR BT  = high-dose-rate brachytherapy; PDR  = pulsed dose rate; 3D-CRT  = three-dimensional conformal radiotherapy; IMRT  = intensity modulated radiotherapy; NR  = not reported; NS  = not significant.

Based on new clinical evidence, the presence of ILC should not influence decisions regarding local therapy, and patients with ILC can be successfully treated with BCS and APBI. Furthermore, small cell LCIS associated with an invasive tumour should not be considered as a contraindication for either breast-conserving therapy or APBI [51].

#### Ductal carcinoma in situ (DCIS)

In 2009, pure DCIS was included in the intermediate-risk group, since that time, most earlyAPBI trials excluded patients with DCIS [12]. Since then, mature APBI studies, using interstitial or other modalities have also included DCIS (Table 5).

**Table 5:** Local recurrence after APBI in patients with DCIS versus invasive carcinoma.

Trial/Author	Type of study	Median FUP (ys)	Technique	N^o^ of patients	Histology		N^o^ of LR according to histology		Higher RR for DCIS?
					Inv. ca.	DCIS	Inv. ca.	DCIS	
NSABP B-39 [22]	Prospective phase 3	10	HDR BT/3D-CRT	2.089	1575	514	58 (3.7%)	32 (6.2)	No (p = NS)
GEC-ESTRO [25]	Prospective phase 3	10	PDR/HDR BT	633	597	36	20 (3.3%)	1 (2.8%)	No (p = NS)
Florence Univ. [20]	Prospective phase 3	5	IMRT	260	237	23	3 (1.3%)	0 (0%)	No (p = 0.84)
Vicini [43]	Retrospective	4.7	HDR BT/ MammoSite/ 3D-CRT	1.598	1.298	300	41 (3.2%)	9 (3.0%)	No (p = 0.77)
Beitsch [58]	Prospective registry trial	5	MammoSite	1.449	1.255	194	42 (3.3%)	8 (4.1%)	No (p = 0.67)
Silverstein [59]	Prospective registry trial	5.2	Xoft 50 kV IORT	1.400	1106	294	48 (4.3%)	16 (5.4%)	No (p = NR)
Shaikh [60]	Retrospective	4.1	IMRT/ELE/ 3D-CRT	312	244	68	6 (2.5%)	3 (4.4%)	No (o = 0.69)
McHaffie [61]	Retrospective	5	HDR BT/ MammoSite	136	104	32	5 (4.8%)	0 (0%)	No (p = 0.24)
Shah [62]	Retrospective	5	IMRT/ELE/ 3D-CRT	331	307	24	7 (2.3%)	0 (0%)	No (p = NR)
Aristei [63]	Prospective phase 2	8	HDR BT	240	224	16	6 (2.7%)	2 (12.5%)	Yes (p = NR)
Goldberg [64]	Meta-analysis	8.6	Mixed	7.329	6.624	705	346 (5.2%)	45 (6.4%)	No (p = 0.85)
All studies					13.571	2.206	582 (4.3%)	116 (5.3%)	

*Abbreviations*

APBI  = accelerated partial breast irradiation; DCIS  = ductal carcinoma in situ; FUP  = follow-up period; LR  = local recurrence; RR  = relative risk; HDR BT  = high-dose-rate brachytherapy; PDR  = pulsed dose rate; 3D-CRT  = three-dimensional conformal radiotherapy; IORT  = intraoperative radiotherapy; IMRT  = intensity modulated radiotherapy; ELE  = electrons; NR  = not reported; NS  = not significant.

In the NSABP-RTOG B-39 trial, 1.031 out of 4.216 patients (24%) had pure DCIS [22]. Although, a slightly higher rate of LR was observed in the DCIS subgroup compared to patients with invasive carcinoma (Table 5), the treatment itself (e.g. APBI versus WBI) had no significant impact on local tumour control. In the DCIS subgroup, at a median follow-up of 122 months, 61 LRs were reported, 32 out of 514 (6.2%) in the APBI arm and 29 out of 498 (5.8%) in the WBI arm, yielding a 10-year cumulative incidence of 6.0 versus 6.5%, respectively with a hazard ratio of 1.01 (95% CI: 0.61–1.68), and with a p-value of 0.28 for interaction. In the DCIS group, 148 (14%) were classified as G1, 260 (25%) as G2, and 289 (28%) as G3, unknown in 334 (32%), and no differences between any of the subgroups for DCIS were found.

In the APBI arm of the GEC-ESTRO phase 3 trial, 36 out of 633 patients (6%) had DCIS [25]. With a median follow-up of 124 months, 21 LRs (3.3%) were observed in the APBI arm, but only one out of 36 patients (2.8%) belonged to the DCIS subgroup, while in the WBI arm, there were 24 patients with pure DCIS and also only one (4.2%) developed LR (p = 0.41).

In the Florence University trial using IMRT in the APBI arm, 23 out of 260 patients (8.8%) had DCIS, and at five years there were only three LRs (1.2%) in the APBI arm, but none in the DCIS subgroup [20].

In a pooled retrospective analysis of Vicini et al [43], 300 out of 1.598 patients (19%) treated with different techniques of APBI had DCIS. The 5-year actuarial IBTR rate was 3.1% for patients with invasive carcinoma versus 2.6% for women with DCIS (p = 0.41). The authors conluded that their results provide further support for considering the inclusion of DCIS to the suitable groupin the ASTRO (American Society for Radiation Oncology) consensus panel guideline.

Similarly, in the American Society of Breast Surgeons (ASBS) MammoSite® registry trial, 194 out of 1.449 patients (13%) were treated with balloon-based APBI [58]. At a median follow-up of five years, the actuarial rate of IBTR was 3.6% for invasive breast cancer and 3.4% for DCIS (p = 0.67).

In a prospective study published by Silverstein et al [59], 1.400 patients were included, 1.106 (79%) with invasive disease and 294 (21%) with DCIS. Patients were treated with IORT using the Xoft Axxent Electronic Brachytherapy System® delivering a single fraction of 20 Gy with 50 kV x-ray irradiation. The 5-year probability of any event (including local, regional or distant recurrence or death) was 6.0% for the 1.175 patients who received IORT only, while it was 6.8% for the DCIS subgroup.

In other smaller retrospective APBI series, LR rate was similar for patients with invasive and non-invasive breast cancers [60], [61], [62]. Only the prospective phase 2 study of Aristei et al [63] reported higher LR rate in patients with DCIS treated with multicatheter HDR BT. Sixteen out of 240 patients (6.7%) had DCIS. However, due to the low number of patients, these data did not provide evidence as to whether or not APBI should be recommended for patients with DCIS.

In the meta-analysis of trials of partial breast irradiation (PBI) published in 2023 by Goldberg et al [64], eleven of the published PBI trials included only patients with invasive disease, and four trials also included patients with DCIS. In these four trials, a total of 1.392 patients with DCIS were randomly assigned to either PBI or WBI. Forty-five out of 705 (6.4%) and 36 out of 687 (5.2%) patients developed LR after PBI and WBI, respectively, with a non-significant adjusted risk ratio of 1.63 (95% CI: 1.00–2.67). In the PBI group, histology (invasive vs DCIS) had no significant effect on the rate of LR (see Table 5).

Thus, based on the latest clinical evidence, APBI can be offered as a part of routine clinical practice for patients with pure DCIS up to 3 cm and with free resection margins ≥2 mm.

#### Histologic grade (HG)

The value of HG as a prognostic factor for LR is also controversial. Clarke et al [65] found that high grade was a strong predictor for LR. On the contrary, in the Hungarian boost trial, HG had no significant effect on local tumour control (LTC), but for grade 3 tumours, the mean time to LR was significantly shorter [66], [67]. However, these data suggest that poorly differentiated malignant cells remaining in the breast after the excision of high-grade tumours generally regrow more rapidly than well- differentiated cells found in low-grade tumours. However, high-grade tumours do not spread more widely through the ductal tree than low-grade carcinomas. In contrast, high-grade tumours tend to regrow more rapidly (if residual cancer has been left by the surgeon). That is the reason why we believe, that grade is not an issue if we consider the target volume to be treated (e.g. partial versus whole breast). Based on these considerations, most APBI studies included tumours with any HG and treated them with consecutive adequate LTC. In the large, randomized APBI clinical trials, 8 to 27% of enrolled patients had grade 3 tumours [19], [20], [21], [22], [23], [24], [25]. Therefore, high-grade breast carcinomas can be treated successfully with APBI.

#### Pathological tumour size (pT)

Although in the NSABP B-06 trial, patients with pT2 tumours were more likely to develop LR following BCS without RT, in most series, tumour size did not significantly influence the LTC following BCS with RT [66], [67], [68], [69]. This is consistent with the pathology data reported by Holland et al [70], which indicate that the microscopic spread beyond the primary tumour is similar in pT1 and pT2 tumours.

In most of contemporary APBI series, maximum tumour size was limited to 3 cm [42]. Some investigators experienced that in the case of large-volume (>160 cm^3^) interstitial BT implants, the implant volume (V100) and high-dose regions (V150 and V200) may be associated with a higher incidence of late soft tissue toxicity (e.g. fat necrosis) [[69], [70], [71], [72]]. Investigators of the RAPID trial, who used external beam 3D-CRT technique in the APBI arm, reported that late radiation toxicity and adverse cosmesis occurred more frequently in patients treated with APBI, which can be attributed to the large treated volume with a median V95 of 332 cm^3^ [23]. Based on these clinical observations, large pT2 (>3 cm) and pT3-4 tumours are not suitable for APBI.

#### Surgical margin status

Positive margin status is generally accepted as a major risk factor for LR after BCS and RT [66], [73], [74], [75]. Furthermore, the number of positive margins, as well as the width of clear surgical margins, significantly influence LTC [66], [74], [75]. The acceptance of the necessary resection margin after BCS has changed significantly over the past 40 years. In the pioneering breast conservation trial, Veronesi et al [2] used quadrantectomy to ensure wide clear margins during BCS, but they did not determine the microsopical margin width. The NSABP-B06 trial required a microscopically clear margin, defined as no ink on tumour [1]. Later, the initial inclusion criteria of the EORTC-22881-10882 “boost versus no boost” study (patient enrollment between 1989 to 1996) still required a macroscopically free resection margin of 1–2 cm with microscopically complete excision defined as a negative margin for the invasive component [3], [76]. However, in a retrospective subgroup analysis of 1.616 patients in this trial, positive margins were found in 3.4% and 14% of the patients for invasive and in situ cancer, respectively [76]. On the contrary, the authors found that margin status (negative, close, or positive) had no significant influence on the 10-year LR rate.

In the advent of APBI, investigators used conservative patient selection criteria [6], [7], [8]. Therefore, in most phase 3 APBI trials (excluding IORT trials with unknown margin status at randomisation), at least 2 mm microscopically free margin was needed [18], [19], [20], [21], [24], [25], [26], [27]. Only two studies (RAPID, and NSABP B-39) allowed patients with close (<2 mm) resection margins, but neither the number of patients with close resection margins, nor the LR rates according to margin status (e.g. close vs. clear) were reported separately [22], [23]. Since there is a lack of clinical evidence on the efficacy of APBI in patients with close surgical margins, the original GEC-ESTRO recommendation classified these patients as being in the intermediate-risk category, while also considering them to be potential candidates for APBI, and stating that their treatment should be conducted within the framework of prospective clinical trials [12].

In 2013, the Society of Surgical Oncology/American Society for Radiation Oncology (SSO/ASTRO) consensus guidelines stated that the routine practice to obtain negative margin widths wider than no ink on tumour for stage I-II invasive breast cancer is not indicated, as wider margins do not significantly lower IBTR risk [77]. On the contrary, in 2015 the same SSO/ASTRO multidisciplinary margin panel suggested that margins of at least 2 mm in DCIS patients undergoing WBI are associated with a reduced risk of IBTR as opposed to narrower negative margin widths [78].

Several meta-analyses have recently been published that directly addressed the effect of reduced resection margins on LR rate and survival after BCT [79], [80], [81]. The meta-analysis of Houssami et al [79], which included 28.126 patients from 33 studies, confirmed that negative margins reduced the risk of LR, however, increasing the margins was not significantly associated with reduced risk of LR. The meta-analysis of Shah et al [80] from 2020 includes 38 studies that examined 54.502 patients treated with BCS and WBI between 1968 and 2010. Absolute LR rates decreased over time for each margin width cohort, and the impact of margin width on LR rates decreased substantially, with very small (<1%) differences between the narrowest (no tumour on ink) and widest (>5 mm) margin groups. Authors of both studies concluded that the results support the no-tumour-on-ink guideline, and the use of wider margins is unlikely to provide any significant benefit in terms of long-term local control.

In a population-based cohort study conducted in Vancouver that used multivariable and matched-pair analyses, results were reported for 10.863 women treated with BCS and WBI between 2001 and 2011 [81]. The 10-year cumulative incidence of LR in the negative (≥2 mm; n = 9.241), close (<2 mm; n = 1.310), and positive (tumour touching ink; n = 312) margin cohorts was 1.8%, 2.0%, and 1.1%, respectively (p = 0.759), although significantly more patients with close and positive margins received a tumour bed boost after WBI.

The results of a recent systematic reviews and meta-analyses evaluating the impact of resection margin width on LR have confirmed that, in breast conservation for DCIS, a resection margin >2 mm is associated with an approximately 70% reduction in the risk of LR compared with a narrower margin width of 0.1–2 mm [[80], [82]]. They also found that the increased risk of LR was offset through tumour bed boost irradiation in patients with intermediate- and high-grade DCIS with close margins.

Although the direct evidence is limited on the efficacy of APBI for patients with close surgical margins, based on the collective clinical evidence obtained from recent meta-analyses of BCT and WBI, APBI can be offered as a part of routine clinical practice for patients with invasive breast carcinoma excised with close (≤2 mm) but clear margins (e.g. “no tumour on ink”). However, for DCIS patients, APBI can be offered only to patients with clear (≥2 mm) margins.

#### Multifocality, multicentricity

It is evident that patients with multicentric tumours (defined as the presence of separate tumour foci more than 2 cm from the index cancer) should not be treated with APBI, because PBI is unable to cover the extent of disease. On the other hand, several studies, including the largest phase 3 APBI trial, have proved that unicentric but multifocal tumours (defined as separate tumour foci within 2 cm of the index lesion) can be successfully treated with APBI [22], [23], [83], [84], [85].

#### Extensive intraductal component (EIC)

EIC is usually reported when 25% or more of an invasive ductal cancer consists of intraductal carcinoma, and DCIS is also present in the adjacent breast tissue. Holland et al [70], [86] reported that patients with EIC were more likely to have residual tumour beyond 2 cm distance from the reference tumour than patients without EIC (33% versus 2%, respectively). The amount of residual tumour was also correlated with the presence of EIC, which explains why patients with EIC positive tumours were more likely to fail locally following BCS and WBI [66], [87], [88], [89], [90], [91].

In four phase 3 APBI clinical trials (Budapest, Florence, Barcelona, GEC-ESTRO), EIC was an exclusion criterion [18], [20], [21], [24], [25], [26]. In other studies, patients with EIC were allowed to be treated with APBI, but to date, none of these studies have yet conducted a subgroup analysis to assess the LR rate for patients with EIC positive tumours [19], [22], [23], [27].

According to the results of the ASBS MammoSite APBI trial and another non-randomized study from the University of Wisconsin, among the multiple variables examined for a potential association with ipsilateral breast failure, only the presence of an EIC was associated with the development of a LR [92], [93]. Therefore, EIC is also considered a contraindication for APBI.

#### Hormone- and HER2-receptor status

For patients with triple negative breast cancer (TNBC) and HER2 positive early-stage breast cancer patients, there is insufficient evidence regarding PBI, and most of available data comes from retrospective series, and the number of patients enrolled in phase 3 clinical trials is very small. Since there are currently no randomized controlled trials comparing WBIand PBI treatments in patients currently considered high-risk (i.e. TNBC and HER2+), it is impossible to determine whether oncological outcomes would be worse with PBI than WBI. As a result, current international guidelines have generally not considered TNBC patients suitable for PBI treatment [12], [13], [14], [94], [95].

In triple negative invasive breast cancer, the outcomes of PBI vary. The major phase 3 trials used variable inclusion criteria and did not assess whether PBI following BCS was equivalent or non-inferior to WBI according to different molecular subgroups [18], [19], [20], [21], [22], [23], [24], [25]. None of the large randomized trials excluded estrogen receptor (ER)/progesterone receptor (PR)-negative patients for PBI, however, this patient population generally accounted for only 5–8% of the study cohort. Moreover, the current evidence remains limited, and the results areare contradictory. Although, favourable outcomes are reported from retrospective series with a limited number of patients and potential selection bias, it is a well-known fact that TNBC is associated with higher rates of ipsilateral, regional, and distant metastases recurrence [28], [61], [93], [96], [97], [98], [99]. This, in consideration with other high-risk factors, such as young age or high histological grade, calls for caution, as this subgroup of patients is not suitable for PBI. Since there is currently no evidence from randomized trials for patients with TNBC, , PBI should currently be offered in these cases only within the context of a clinical trial.

Another group of patients consists of those with HER2+ tumours. Until the advent of trastuzumab, HER2+ tumours were considered the most adverse subtype, however, with the development of HER2 directed therapies, this subtype has become one of those with the the most favourable prognosis. Although HER2 positivity was not an exclusion criterion for participation in PBI treatment in phase 3 trials, it is recognized that the number of HER2+ patients enrolled in the trials was very low, and such patients were reported only in the RAPID, IMPORT-LOW and Florence trials, where they accounted for 4.7%, 4% and 2.8% of the total cohorts, respectively [19], [20], [21], [23]. Moreover, none of these studies analysed the specific outcomes associated with HER2+ subtype as a predictor of oncological outcomes.

In this regard, O’Brien et al [100] recently published their results of a prospective study including 52 HER2+ patients treated with external beam APBI (using 40 Gy in ten daily fractions) and adjuvant HER2+ directed therapy plus chemotherapy (in 81% of cases), reporting 3.8% of LRs at two years, without any regional or distant recurrences observed. In addition, the results obtained from the ATEMPT trial, which evaluated the outcomes for early-stage HER2+ tumours treated with adjuvant trastuzumab and taxanes, showed that LR rates werebelow 2% [101], [102], [103]. Based on these data, patients with primary early-stage HER2+ tumours may be considered well suited for de-escalation therapy with PBI instead of WBI.

In summary, given the limited evidence on PBI in patients with TNBC, this subgroup of high-risk patients should not be considered for PBI, except within the context of a clinical trial. On the other hand, given the change in prognostic paradigm for HER2+ patients secondary to the development of HER2-directed therapies, these patients may be considered as potential candidates for PBI treatment, provided they receive adjuvant HER2 directed therapy. However, HER2+ patients receiving neoadjuvant chemo-biological therapy should not be treated with APBI.

#### Lympho-vascular invasion (LVI)

Peritumoral LVI has been reported by numerous authors as a risk factor for LR after BCS [104], [105], [106]. However, others found that LVI was not an independent predictor of LR on multivariate analysis, as patients with LVI had a younger median age, were more often pre- or perimenopausal, and had T2, physically palpable, invasive ductal, node positive, and grade 3 tumours, compared with patients without LVI [107].

Based on the assumption that, in the presence of LVI, malignant cells can spread widely in the breast via lympho-vascular spaces, the original GEC-ESTRO recommendation favoured a conservative approach, and recommended APBI only for patients without LVI [12]. In the Budapest and GEC-ESTRO phase 3 APBI trials, LVI was a contraindication [18], [24], [25]. Although LVI was permitted in the majority of other randomized APBI trials, the majority of patients who participated did not have LVI. In most of these studies, due to the low incidence rates, it was not possible to conduct a meaningful statistical analysis of the effect of LVI in the patient subgroups treated with PBI or WBI [18], [24], [25]. In the IMPORT-LOW and Florence University trials, 7% of eligible patients had LVI, and the presence of LVI did not increase the risk of LR following PBI (Table 6) [19], [20]. In other prospective and retrospective series of APBI, conflicting results have been reported regarding the efficacy of APBI in patients with LVI positive tumours (see Table 6) [56], [93], [108], [109].

**Table 6:** Local recurrence after APBI for patients with LVI+ tumours.

Author/Trial	Type of study	Median FUP (months)	Technique	N^o^ of patients	LVI		N^o^ of LR according to LVI		Higher RR for LVI?
					Yes	No	LVI+	LVI-	
IMPORT-LOW [19]	Prospective	72	IMRT	494*	35 (7%)	459 (93%)	0 (0%)	6 (1%)	No (p = NR)
Florence Univ. [20]	Prospective	60	IMRT	260	19 (7%)	241 (93%)	0 (0%)	3 (1%)	No (p = NR)
Gabani P [108]	Prospective	120	HDR BT	175	9 (5%)	166 (95%)	NR	NR	Yes (p = 0.037)
Philippson C [109]	Prospective	72	IORT ELE	996	66 (7%)	930 (93%)	NR	NR	No (p = 0.921)
Cannon MD [93]	Retrospective	61	HDR BT	277	20 (7%)	257 (93%)	3 (15%)#	10 (4%)#	Yes (p = 0.016)
Mills MN [56]	Retrospective	60	HDR BT	946	43 (5%)	903 (95%)	1 (2%)	17 (2%)	No (p = 0.907)

*Abbreviations*

APBI  = accelerated partial breast irradiation; WBI  = whole breast irradiation; LVI  = lympho-vascular invasion; FUP  = follow-up period; LR  = local recurrence; RR  = relative risk; HDR BT  = high-dose-rate brachytherapy; IMRT  = intensity modulated radiotherapy; IORT  = intraoperative radiotherapy; ELE  = electron; NR  = not reported; NS  = not significant. *Among the 669 partial breast patients, 494 patients had data on LVI. # locoregional recurrence rate (not LR).

In a recent clinico-pathological study of the Memorial Sloan Kettering Cancer Center, the 5-year locoregional recurrence (LRR) rate was significantly affected by the extent of LVI [106]. The cumulative 5- and 10-year incidence of LRR was 9.6% and 13% in patients with extensive LVI, which was significantly higher than those observed in the focal/suspicious LVI group (6.6% and 9.9%, respectively). However, the corresponding rates of LRR were lowest in the LVI-negative group (4.0% and 6.8, respectively). Based on these new findings and considerations, in case of focal LVI (e.g. if LVI is present in a single block only), APBI can be considered a cautious but appropriate treatment option.

#### Surgical nodal staging – pathologic axillary status (pN)

In the majority of early APBI trials, in which surgical nodal staging was incomplete (or fully omitted), a high incidence of LR was reported [110], [111], [112]. Therefore, candidates for APBI should undergo surgical axillary staging.

The eligibility of patients with positive lymph nodes (pN+) for APBI is controversial, as prospective data are limited. In some studies, including the NSABP-B39 Phase 3 trial, women with fewer than four affected axillary lymph nodes with or without extracapsular extension were also considered for APBI. [22], [83], [84], [113]. Other studies (including successful European APBI studies) selected only patients whose lymph nodes were negative or not more than microscopically involved [6], [7], [18], [24], [114].

A literature review from the past 15 years has identified several prospective trials involving pN+ cases [[19], [20], [21], [22], [24], [25], [28], [115]]. In the NSABP-B39 trial, 218 patients with one to three positive lymph nodes were treated with APBI [22]. Following APBI, the 10-year cumulative incidence of LR in the pN1a subgroup was 4.7%, compared to 2.8% for WBI, however, the difference was not significant. In the ELIOT trial, 26% of patients (n = 169) in the APBI group had one to three (pN1a) or more than 4 (pN2a) positive axillary nodes. The 15-year LR rate for node negative, pN1a, and pN2a patients was 11.4%, 14.1%, and 24.2%, respectively, and the difference was significant (p = 0.026; RR: 2.63) for patients with four or more positive lymph nodes [28]. In the Florence University trial, only 19 patients were treated with APBI, and nodal status had no impact on oncological outcome [21].

A retrospective study by Sumodhee et al [53] did not find that nodal status influenced the oncological outcomes. Nonetheless, the results of a study published by Kamrava et al [44] that pooled the data from multiple registries, 5-year distant metastasis-free survival and cancer-specific survival were worse in pN+ patients than in pN0 patients. There was no significant difference in oncological outcomes between the pN1a and pN1mi cases, however, the 5-year LR rates were 5.1% and 0%, respectively. The William Beaumont group analysed the impact of lymph node status on clinical outcome in order to compare the results of 471 node-negative and 39 node-positive patients [53]. There was no significant difference in the LR rate between the two groups. However, significantly more node-positive patients developed regional (6.1% vs. 0%; p<0.001) and distant recurrence (8.9% vs. 2.2%; p = 0.005). Nevertheless, none of the studies reported a statistically significant influence of pN1mi on oncological outcomes. Therefore, we recommend that APBI be used only for patients with pN0 or pN1mi axillary status confirmed by axillary surgical staging (either with sentinel lymph node biopsy or axillary lymph node dissection).

Recent 5-year results of the SOUND and INSEMA trials suggest that omitting axillary surgery is a safe option for selected elderly patients with clinical stage I (cT1 cN0) luminal (ER and HER2 positive) breast cancer [116], [117]. Although, these patients are often suitable candidates for APBI, the original GEC-ESTRO recommendations excluded patients with pathologically unknown (pNx) axillary status [12]. This means, that we must contend with uncertainity and a lack of clinical evidence regarding the indication of APBI in patients who have not undergone axillary surgery (pNx) [118]. Therefore, we recommend that APBI be offered to patients with invasive breast cancer and pNx axillary status exclusively in the context of prospective clinical trials. Therefore, patients treated according to the INSEMA or SOUND protocols are not suitable for APBI and should be treated with WBI.

#### Neoadjuvant chemotherapy

Since there are no studies evaluating the feasibility of APBI following neoadjuvant chemotherapy and BCS, these patients should not receive APBI.

### GEC-ESTRO recommendations on patient selection for APBI

Based on the evidence presented in this revision, the GEC-ESTRO BCWG recommends two categories as guidelines for selecting patients for APBI:

#### Low-risk group

Low-risk patients who meet all the criteria described in Table 7 are eligible for APBI outside the context of prospective clinical trials. For these women, APBI or WBI can be offered as alternative treatment options following BCS in the daily routine practice. This group includes patients aged at least 40 years with unicentric, unifocal or multifocal within 2 cm of the index lesion, pTis, pT1-2 (≤30 mm) pN0 or pN1mi, all histology types of breast cancer without the presence of EIC, without extensive LVI and with negative surgical margins by NSABP criteria (e.g. ”no tumour on ink”) for invasive carcinomas and ≥2 mm for DCIS, and HER2+ patients provided that they are going to receive adjuvant HER2 directed therapy.

**Table 7:** GEC-ESTRO updated recommendations on patient selection for accelarated partial breast irradiation.

Characteristic	A/ Low-risk group	B/ High-risk group
	Good candidates for APBI	Contraindication for APBI
Patient age	>40 years	≤40 years
Histology	All invasive cc. (including ILC)	-
Associated LCIS	Allowed	-
DCIS	Allowed	-
HG	Any	-
Tumour size	pTis-1-2 (≤30 mm)	pT2 (>30 mm), pT3, pT4
Surgical margins	Negative (no tumour on ink) for invasive tumors	Positive for invasive tumors
	≥2 mm for DCIS	<2 mm for DCIS
Multicentricity	Unicentric	Multicentric
Multifocality	Unifocal or multifocal (limited within 2 cm)	Multifocal (>2 cm from the index lesion)
EIC	Not allowed	Present
LVI	Focal allowed	Extensive
HER2 status*	Any	-
Triple negative status	Not allowed	Present
Nodal status	pN0 or pN1mi (by SLNB or ALND**)	pNx; ≥pN1a (by SLNB or ALND**)
BRCA 1–2 gene mutation	Not allowed	Present
Neoadjuvant chemotherapy	Not allowed	If used

*Abbreviations*

APBI  = accelerated partial breast irradiation; IDC  = invasive ductal carcinoma; ILC  = invasive lobular carcinoma; LCIS  = lobular carcinoma in situ; DCIS  = ductal carcinoma in situ; HG  = histologic grade; EIC  = extensive intraductal component; LVI  = lympho-vascular invasion; ER  = estrogen receptor; PR  = progesterone receptor; SLNB  = sentinel lymph node biopsy; *APBI allowed for HER2+ pts. receiving postoperative anti-HER2 systemic therapy; **ALND  = axillary lymph node dissection (at least 6 nodes pathologically examined); BRCA  = BReast CAncer.

#### High-risk group

The high-risk group of women (Table 7) should not be treated with APBI, as there is sufficient evidence against the use of APBI in these patients. These women should be treated with WBI, with or without tumour bed boost, according to the available clinical evidence. This group of patients includes patients with BRCA 1–2 mutations or ageing ≤40 years; having positive margins, and/or multicentric or large (>30 mm), and/or triple negative tumours, and/or EIC positive, and/or extensive LVI, or macrometastatic positive lymph nodes (≥pN1a) or unknown axillary status (pNx). Since there are no clinical studies available that evaluate the feasibility of APBI following neoadjuvant chemotherapy and BCS, these patients were also included in the high-risk group.

## Conclusions

Based on the available evidence from prospective clinical trials that have yielded excellent results in selected patient groups, we recommend that APBI be used outside of clinical trials, provided that strict patient selection criteria are applied including only low-risk early breast cancer and that systematic quality assurance procedures are followed regarding indication and treatment performance [119], [120], [121]. These recommendations provide clinical guidance for physicians and patients to consider APBI as a part of everyday clinical practice. However, it should be noted that in several subgroups (i.e. age 40–50 years, LVI, ILC, multifocality) for which updated recommendations have been issued, the number of patients and the number of local recurrences are quite low, so the statistical certainty is limited. Therefore, shared decision-making with the patients involved remains important in these situtations.

The main objective of the GEC-ESTRO BCWG was to formulate recommendations on patient selection for the use of BT. In this context, we believe that the recommendations given here also apply to other emerging alternative techniques of APBI, including external beam RT and IORT.

*Remarks:* The GEC-ESTRO Breast Cancer Working Group assumes no liability for the information, conclusions, and findings contained in its recommendations. It is also to be noted that adherence to the recommendations will not ensure successful treatment in every situation. The medical decisions regarding any specific therapy must be made by the physician and the patient taking into account all aspects of the medical records presented by the individual patient.

## Declaration of competing interest

The authors declare that they have no known competing financial interests or personal relationships that could have appeared to influence the work reported in this paper.
